# A conserved genetic architecture among populations of the maize progenitor, teosinte, was radically altered by domestication

**DOI:** 10.1073/pnas.2112970118

**Published:** 2021-10-22

**Authors:** Qiuyue Chen, Luis Fernando Samayoa, Chin Jian Yang, Bode A. Olukolu, Alessandra M. York, Jose de Jesus Sanchez-Gonzalez, Wei Xue, Jeffrey C. Glaubitz, Peter J. Bradbury, Maria Cinta Romay, Qi Sun, Edward S. Buckler, James B. Holland, John F. Doebley

**Affiliations:** ^a^Laboratory of Genetics, University of Wisconsin–Madison, Madison, WI 53706;; ^b^US Department of Agriculture–Agricultural Research Service Plant Science Research Unit, North Carolina State University, Raleigh, NC 27695;; ^c^Department of Crop Science, North Carolina State University, Raleigh, NC 27695;; ^d^Department of Entomology and Plant Pathology, University of Tennessee, Knoxville, TN 37996;; ^e^Centro Universitario de Ciencias Biológicas y Agropecuarias, Universidad de Guadalajara, Zapopan, Jalisco CP45110, Mexico;; ^f^Institute of Biotechnology, Cornell University, Ithaca, NY 14853;; ^g^US Department of Agriculture–Agricultural Research Service, Cornell University, Ithaca, NY 14853;; ^h^Genomic Diversity Facility, Cornell University, Ithaca, NY 14853

**Keywords:** maize, teosinte, domestication, evolution, selection

## Abstract

Very little is known about how domestication was constrained by the quantitative genetic architecture of crop progenitors and how quantitative genetic architecture was altered by domestication. Yang et al. [C. J. Yang *et al.*, *Proc. Natl. Acad. Sci. U.S.A.* 116, 5643–5652 (2019)] drew multiple conclusions about how genetic architecture influenced and was altered by maize domestication based on one sympatric pair of teosinte and maize populations. To test the generality of their conclusions, we assayed the structure of genetic variances, genetic correlations among traits, strength of selection during domestication, and diversity in genetic architecture within teosinte and maize. Our results confirm that additive genetic variance is decreased, while dominance genetic variance is increased, during maize domestication. The genetic correlations are moderately conserved among traits between teosinte and maize, while the genetic variance–covariance matrices (*G*-matrices) of teosinte and maize are quite different, primarily due to changes in the submatrix for reproductive traits. The inferred long-term selection intensities during domestication were weak, and the neutral hypothesis was rejected for reproductive and environmental response traits, suggesting that they were targets of selection during domestication. The *G*-matrix of teosinte imposed considerable constraint on selection during the early domestication process, and constraint increased further along the domestication trajectory. Finally, we assayed variation among populations and observed that genetic architecture is generally conserved among populations within teosinte and maize but is radically different between teosinte and maize. While selection drove changes in essentially all traits between teosinte and maize, selection explains little of the difference in domestication traits among populations within teosinte or maize.

## Significance

We investigated the genetic architecture of maize domestication using a quantitative genetics approach. With multiple populations of teosinte and maize, we also compared the genetic architecture among populations within maize and teosinte. We showed that genetic architecture among populations within teosinte or maize is generally conserved, in contrast to the radical differences between teosinte and maize. Our results suggest that while selection drove changes in essentially all traits between teosinte and maize, selection is far less important for explaining domestication trait differences among populations within teosinte or maize.

The question of how the evolutionary forces of natural selection, genetic drift, and mutation shaped the evolutionary potential of traits remains one of the core research areas in modern evolutionary biology (1). Darwin (2) pioneered the use of domestication as a model for natural evolution, and domestication continues to allow the study of the impact of selection on genetic architecture through comparison of predomestication and postdomestication populations (3, 4). During the past several decades, plant geneticists have focused on analyzing the genetic control of the morphological differences between crops and their progenitors by analyzing inheritance in wild-by-cultivated mapping populations. With the rapid development of sequencing and omics technologies, large-scale population genetic studies have been performed to study genomic, transcriptomic, and metabolic changes associated with crop domestication (5). However, how the genetic architecture among wild species influenced and was altered by domestication is largely unknown. Recently, Yang et al. (6) reported on the constraints imposed by, and changes under domestication of, the genetic architecture of a suite of morphological traits in a single sympatric pair of teosinte and landrace maize.

In this paper, we evaluated whether the inferences reported by Yang et al. (6) are consistent across a broader sample of paired wild and domesticated populations, again using teosinte and maize as a model system. We observed that additive genetic variance is decreased, while dominance genetic variance is increased, during maize domestication. The genetic correlations are moderately conserved among traits between teosinte and maize, while the ***G***-matrices of teosinte and maize are quite different, primarily due to changes in the submatrix for reproductive traits. The ***G***-matrix in teosinte placed considerable constraint on selection during the early domestication process, which became even greater along the domestication trajectory. We observed weak selection intensities during domestication, but rejected the hypothesis of neutral changes in reproductive and environmental response traits. Further, the genetic architecture among populations within teosinte or maize is generally conserved in contrast to the radical differences between teosinte and maize. While selection drove changes in essentially all traits between teosinte and maize, selection is far less important for explaining trait differences among populations within teosinte or maize. We observed little or no evidence for adaptive divergence among teosinte populations for the traits we assayed, a result that stands in contrast to observations in other wild plant species (1).

## Results

**Fig. 1.**
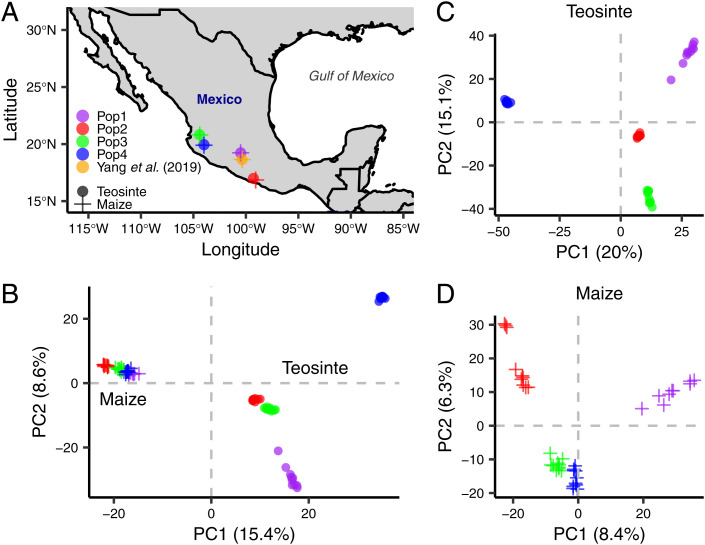
Parental information for four teosinte populations and four matching maize populations. (*A*) Teosinte and maize landrace populations were collected from the states of Michoacán (Pop1), Guerrero (Pop2), Nayarit (Pop3), and Jalisco (Pop4) in Mexico. Each maize landrace population was sampled at a location near the corresponding teosinte population. (*B*) PCA of genotyping data for teosinte and maize parents together. (*C*) PCA for teosinte parents only. (*D*) PCA for maize parents only. PC1 and PC2 are plotted with the percentage of variance explained.

To better understand how the genetic architecture influenced and was altered by maize domestication, we analyzed 16 traits (Table 1) in 4 pairs of teosinte and maize landrace populations, where each pair represents a localized geographic collection. Each of the 8 populations was created by selfing and intermating samples of 10 parents, resulting in 5,101 progeny in teosinte and 4,641 progeny in maize. The collection sites represent a wide geographical range from the states of Michoacán, Guerrero, Nayarit, and Jalisco in Mexico, hereafter referred to as Pop1, Pop2, Pop3, and Pop4, respectively (Fig. 1*A*). Genome-wide single-nucleotide polymorphism (SNP) markers were obtained for both parents and progeny in each population. Principal Component Analysis (PCA) of genotyping data of the parents shows that there is strong genetic differentiation among the four teosinte and four maize landrace populations (Fig. 1 *B–D*). Phenotypic data of the two subspecies were collected in adjacent field blocks over 2 y. In this study, we focus on comparing our results using this broad sample with those of Yang et al. (6), who studied a single pair of populations sampled from near the town of Palmar Chico in the Estado de México, west of Mexico City. Additionally, we assess variation among populations within both teosinte and maize.

**Table 1.** Trait abbreviations

Trait	Acronym	Units	Trait group
Days to anthesis	DTA	Days	Vegetative/Flowering Time
Days to silking	DTS	Days	Vegetative/Flowering Time
Plant height	PLHT	Centimeters	Vegetative/Flowering Time
Leaf length	LFLN	Centimeters	Vegetative/Flowering Time
Leaf width	LFWD	Centimeters	Vegetative/Flowering Time
Tiller number	TILN	Count	Environmental Response
Prolificacy	PROL	Count	Environmental Response
Lateral branch node number	LBNN	Count	Environmental Response
Lateral branch length	LBLN	Millimeters	Environmental Response
Lateral branch internode length	LBIL	Millimeters	Environmental Response
Ear length	EL	Millimeters	Reproductive
Cupules per row	CUPR	Count	Reproductive
Ear diameter	ED	Millimeters	Reproductive
Grains per ear	GE	Count	Reproductive
Ear internode length	EILN	Millimeters	Reproductive
Grain weight	GW	Milligrams	Reproductive

List of 16 teosinte–maize landrace comparable traits and the corresponding acronyms, units, and trait groups.

### Univariate Analysis.

#### There is a higher level of heritable variation (*h^2^*) in teosinte than maize landrace, with reproductive traits showing the strongest reduction from teosinte to maize.

**Fig. 2.**
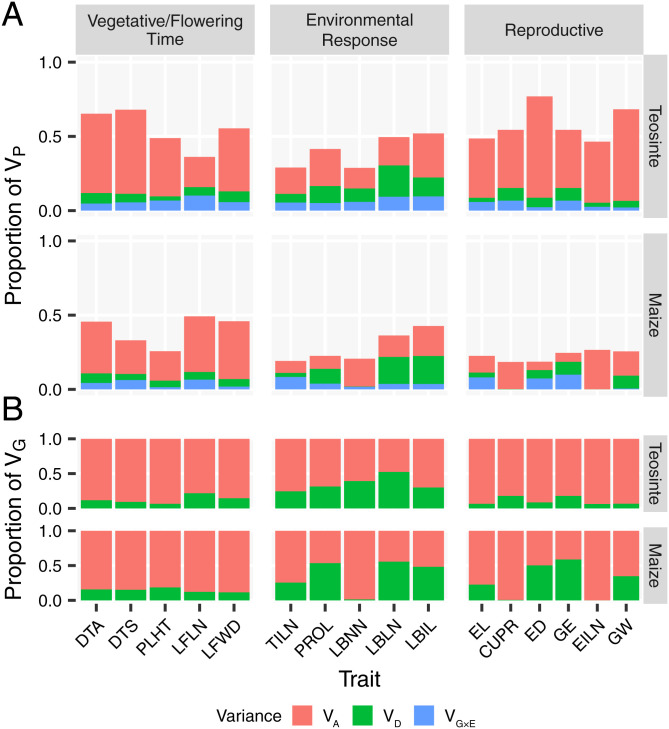
Variances for 16 teosinte and maize landrace comparable traits. (*A*) Proportions of phenotypic variance (*V_P_*) attributed to additive genetic variance (*V_A_*), dominance genetic variance (*V_D_*), and genetic-by-environment variance (*V_GxE_*). (*B*) Proportions of genetic variance (*V_G_*) attributed to additive genetic variance (*V_A_*) and dominance genetic variance (*V_D_*).

By fitting a common univariate linear mixed model, the phenotypic variance (*V_P_*) was decomposed into genetic variance (*V_G_*), which includes additive genetic variance (*V_A_*) and dominance genetic variance (*V_D_*), genetic-by-environment interaction variance (*V_G×E_*), and environmental variance (*V_E_*). A single model was applied to the four combined teosinte populations, and a separate model was applied to the four combined maize populations. Each model produced variance component estimates jointly estimated across all populations within a subspecies, which include variation among as well as within populations (Dataset S1). The narrow-sense heritability was calculated as *h*^2^ = *V_A_*/*V_P_*. We observed higher *h*^2^ in teosinte (*h*^2^ = 0.38, ranging among traits from 0.14 to 0.68) than in maize landrace (*h*^2^ = 0.19, ranging from 0.06 to 0.39) (Fig. 2*A* and *SI Appendix*, Table S1). We grouped the 16 traits into three previously defined groups: Vegetative/Flowering Time, Reproductive, and Environmental Response traits (6). Among the three trait groups, Reproductive traits showed the strongest depletion in *h*^2^ from teosinte (*h*^2^ = 0.48) to maize landrace (*h*^2^ = 0.14), followed by Vegetative/Flowering Time and Environmental Response traits (Fig. 2*A* and *SI Appendix*, Table S1). Of the 16 traits, ED (see Table 1 for trait abbreviations) showed the most depletion in *h*^2^ from teosinte (*h*^2^ = 0.68) to maize landrace (*h*^2^ = 0.06) (Fig. 2*A*). Overall, our results confirmed the observations from Yang et al. (6), but with one difference—PROL showed significantly larger variances (*V_A_*, *V_D_*, and *V_G×E_*) in maize landrace in this study (*SI Appendix*, Fig. S1).

We also compared the four teosinte populations to their four matched maize populations by estimating the genetic and phenotypic variances separately for each population. We observed similar levels of depletion in *h*^2^ from teosinte to maize landrace in separate individual populations (*SI Appendix*, Figs. S2 and S3 and Dataset S1). Average *h*^2^ across all traits decreased from 0.30, 0.34, 0.34, and 0.34 in teosinte to 0.18, 0.16, 0.16, and 0.20 in maize landrace for Pop1, Pop2, Pop3, and Pop4, respectively (*SI Appendix*, Table S1). Among the three trait groups, Reproductive traits also showed the strongest depletion in *h*^2^ from teosinte to maize landrace in all four individual populations (*SI Appendix*, Table S1). Taken together, our observations indicate that there was a depletion in additive genetic variance and narrow-sense heritability during maize domestication, especially for Reproductive traits.

#### While additive genetic variance (*V_A_*/*V_P_*) declined in maize landrace relative to teosinte, the proportion of genetic variance attributable to dominance (*V_D_/V_G_*) increased, with Reproductive traits showing the most increase.

We observed lower *V_D_*/*V_G_* in teosinte (*V_D_*/*V_G_* = 0.19, ranging from 0.06 to 0.52) than maize landrace (*V_D_*/*V_G_* = 0.27, ranging from 0.00 to 0.59) (Fig. 2*B* and *SI Appendix*, Table S1) when the populations were analyzed jointly. Among the three trait groups, Reproductive traits showed the most increase in *V_D_*/*V_G_* from teosinte (*V_D_*/*V_G_* = 0.11) to maize landrace (*V_D_*/*V_G_* = 0.28), while Vegetative/Flowering Time and Environmental Response traits showed little difference in *V_D_*/*V_G_* between teosinte and maize landrace (*SI Appendix*, Table S1). Of the 16 traits, ED showed the most increase in *V_D_*/*V_G_* from teosinte (*V_D_*/*V_G_* = 0.09) to maize landrace (*V_D_*/*V_G_* = 0.50) (Fig. 2*B* and Dataset S1). These results are similar to those of Yang et al. (6), indicating that, as additive variance was depleted during domestication, dominance variance became a greater proportion of the total genetic variance.

We observed similar trends of higher *V_D_*/*V_G_* in maize landrace than teosinte when the four matching pairs of individual populations were analyzed separately (*SI Appendix*, Figs. S2 and S3). *V_D_*/*V_G_* increased from 0.17, 0.20, 0.16, and 0.13 in teosinte to 0.30, 0.36, 0.40, and 0.31 in maize landrace for Pop1, Pop2, Pop3, and Pop4, respectively (*SI Appendix*, Table S1). Indeed, the amount of within-population increase between maize and teosinte is even more than that in combined populations. Among the three trait groups, Reproductive traits also showed the most increase in *V_D_*/*V_G_* from teosinte to maize landrace in Pop1 and Pop2 (*SI Appendix*, Table S1).

#### Changes in the apportionment of gene-by-environment interaction variance (*V_G×E_/V_P_*) indicate that maize evolved a different strategy to respond to environmental variation.

Overall, *V_G×E_*/*V_P_* is roughly equal in teosinte (*V_G×E_*/*V_P_* = 0.059, ranging from 0.01 to 0.10) to maize landrace (*V_G×E_*/*V_P_* = 0.048, ranging from 0.02 to 0.10) (Fig. 2*A* and *SI Appendix*, Table S1). However, among the three trait groups, Reproductive traits showed a slight increase in *V_G×E_*/*V_P_* from teosinte (*V_G×E_*/*V_P_* = 0.043) to maize landrace (*V_G×E_*/*V_P_* = 0.064), while Vegetative/Flowering Time and Environmental Response traits showed a slight decrease in *V_G×E_*/*V_P_* from teosinte (*V_G×E_*/*V_P_* = 0.066 and 0.070) to maize landrace (*V_G×E_*/*V_P_* = 0.040 and 0.042) (*SI Appendix*, Table S1).

We observed these same trends when the four matching pairs of individual populations were analyzed separately (*SI Appendix*, Figs. S2 and S3). Reproductive traits consistently showed an increase in *V_G×E_*/*V_P_* from teosinte to maize landrace, while Environmental Response traits showed a decrease in *V_G×E_*/*V_P_* from teosinte to maize landrace in all four individual populations (*SI Appendix*, Table S1). These results are consistent with the observations from Yang et al. (6) and suggest that teosinte responded to environmental variation largely through variation in Vegetative/Flowering Time and Environmental Response traits, while maize landrace shifted to respond to environmental challenges more by variation in Reproductive traits.

#### The estimates of directional selection intensity (*i*) during domestication are on the low end of values of *i* reported under natural selection, with Reproductive traits showing the highest values.

**Fig. 3.**
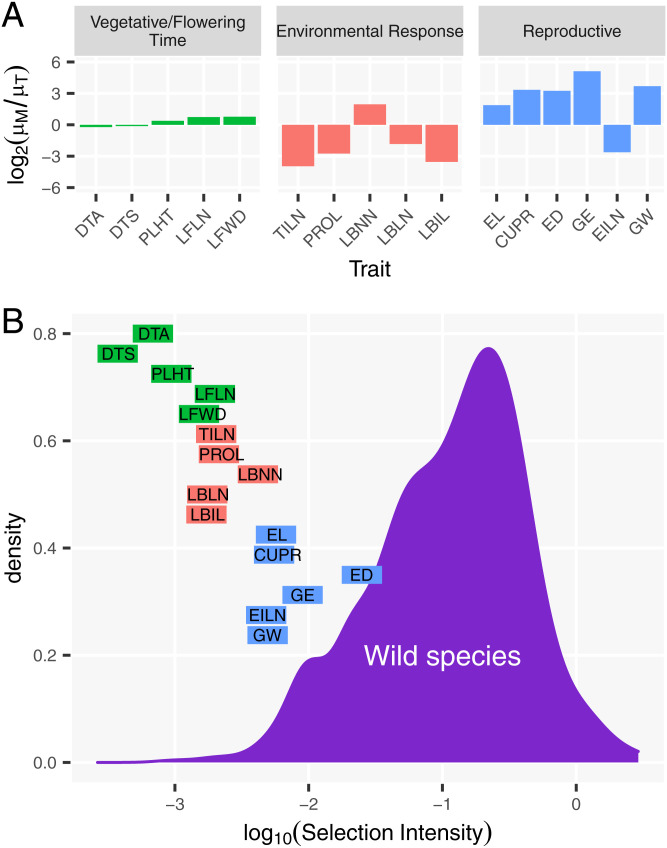
Changes in trait means and selection intensities. (*A*) Changes in trait mean (μ) are measured as $log_{2}\left(\mu_{M}/\mu_{T}\right)$ or fold change of maize landrace outcross mean ($\mu_{M}$) over teosinte outcross mean ($\mu_{T}$). (*B*) Absolute selection intensities (∣i∣) for 741 traits in wild species under natural selection are shown in a density plot [data from Kingsolver et al. (7)], and ∣i∣ for 16 traits in maize under domestication are shown in horizontal bars. The left end of the bars represents selection intensities estimated from 9,000 generations of selection, and the right end represents selection intensities estimated from 4,500 generations of selection.

We estimated selection intensity for each trait and observed weak *i* across all traits (Fig. 3 and *SI Appendix*, Table S2). Among the three trait groups, Reproductive traits showed the highest magnitude of *i* (|*i*| = 0.0034 to 0.02), Environmental Response traits a moderate magnitude of *i* (|*i*| = 0.0012 to 0.003), and Vegetative/Flowering Time traits a low magnitude of *i* (|*i*| = 0.0003 to 0.001). DTA, DTS, and PLHT are the three traits with the lowest magnitude of *i*, suggesting that these traits were under no or weak selection. ED has the largest magnitude of *i*, suggesting that it was strongly selected during maize domestication.

The estimates of selection intensity from separate individual populations are also small with similar trends; however, the Reproductive traits showed much higher magnitude of *i* among three of the matched individual populations, suggesting larger estimates of domestication selection for Reproductive traits in these subpopulations (*SI Appendix*, Fig. S4 and Table S3). Pop4 is an exception, where Reproductive traits show a moderate magnitude of *i*. We should note that Pop4 is highly inbred in teosinte, and it was included in our study for this reason, since we plan to evaluate the importance of inbreeding in a future publication. Overall, the estimates of selection intensity are similar to those of Yang et al. (6).

#### Reproductive and Environmental Response traits show strong evidence of directional selection across all populations, while Vegetative/Flowering Time traits show some, but much weaker, evidence of selection across populations.

**Fig. 4.**
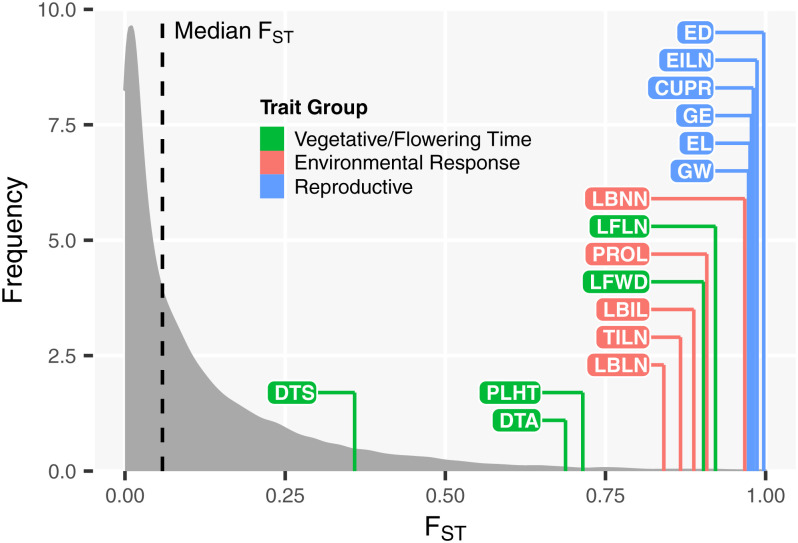
Univariate *Q_ST_*–*F_ST_* comparison for all 16 traits. The gray distribution of *F_ST_* was estimated from 28,081 markers that are in common between teosinte and maize landrace. The *Q_ST_* for each trait is shown as an individual line along the horizontal axis and is colored according to trait group.

We performed univariate *Q_ST_*–*F_ST_* tests for individual traits to ask whether neutral evolution during domestication can be rejected. Among the 16 traits, DTS was the only trait that did not reject the null hypothesis of selective neutrality (Fig. 4 and *SI Appendix*, Table S4). Among the four pairs of matched individual populations, Vegetative/Flowering Time traits (DTA, DTS, and PLHT) did not reject the null hypothesis of selective neutrality in some populations; Reproductive traits were consistent with nonneutrality; and Environmental Response traits seemed to be nonneutral, with the exception of one trait (LBLN) in one population (*SI Appendix*, Fig. S5 and Table S5). Yang et al. (6) rejected the neutral drift model for all traits from a single pair of populations. Our finding for the Vegetative/Flowering Time traits may be more representative, given that we used a broader and more representative sample of teosinte and maize.

### Multivariate Analysis.

#### The genetic correlations among traits are well-conserved between teosinte and maize landrace, with the strongest conservation within Vegetative/Flowering Time traits.

**Fig. 5.**
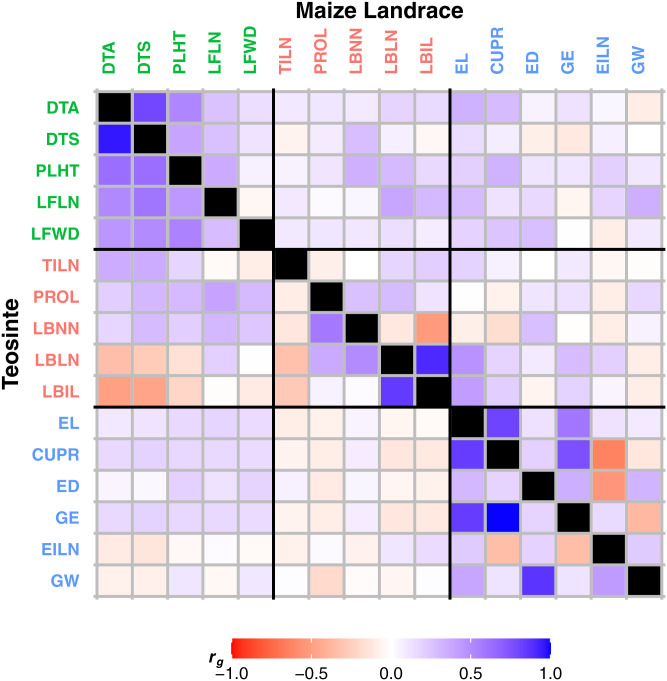
Genetic correlations for 16 teosinte and maize landrace comparable traits. Genetic correlations for traits in teosinte are shown in the bottom left triangle of the matrix, and maize landraces are shown in the top right triangle of the matrix. Genetic correlations are colored according to the scale on the bottom.

By fitting a common bivariate linear mixed model, we estimated the additive genetic correlation (***r_g_***) between each pair of traits in the four combined teosinte populations and the four combined maize populations, respectively. By comparing the genetic correlation matrices between teosinte and maize landrace using the Mantel test (8), our results showed that the genetic correlations for teosinte are overall correlated with those for maize landrace (*r* = 0.49; *P* < 0.0001) (Fig. 5). The genetic correlations are better conserved within the submatrices of each trait group, with the strongest conservation of genetic correlations within the Vegetative/Flowering Time trait group (*r* = 0.88; *P* = 0.06), followed by the Reproductive trait group (*r* = 0.75; *P* = 0.005) and the Environmental Response trait group (*r* = 0.51; *P* = 0.2) (Fig. 5). We note that the correlation for Vegetative/Flowering Time traits is only marginally significant, while the correlation for Environmental Response traits is not significant.

We also compared eigenstructure of the teosinte and maize landrace genetic correlation matrices by calculating the angle between the eigenvectors. Our results showed that the first two leading eigenvectors of the full genetic correlation matrices are 51.8° and 56.7° apart, respectively. For the three trait groups, the submatrices differed by 15.5° and 35.3° for the Vegetative/Flowering Time submatrices, 57.4° and 44.8° for the Environmental Response submatrices, and 26.7° and 83.7° for the Reproductive submatrices. These comparisons suggest that the genetic correlations are well-conserved for Vegetative/Flowering Time traits, but have changed for Reproductive traits and Environmental Response traits.

We observed similar results when comparing the four separate teosinte populations to their four matched maize populations for the genetic correlation matrices (*SI Appendix*, Fig. S6 and Table S6). The Mantel test results showed that the genetic correlation matrices are conserved between teosinte and maize landrace, and they are more conserved within the submatrices of each trait group in all four individual populations. The eigenstructure comparisons showed that the full genetic correlation submatrices are generally well-conserved for Vegetative/Flowering Time traits, but have changed substantially for Reproductive traits and Environmental Response traits.

#### The genetic variance–covariance (*G*) matrices of teosinte and maize landrace are quite different, primarily due to changes in the submatrix for Reproductive traits.

We tested whether the ***G***-matrices (Dataset S2) are conserved between teosinte and maize landrace by comparing the predicted evolutionary responses using Random Skewers (9). Overall, the predicted evolutionary responses are not significantly correlated (*r* = 0.15; *P* = 1.00), suggesting that teosinte and maize landrace ***G***-matrices are quite different. The dissimilarity of ***G***-matrices between teosinte and maize landrace is primarily due to changes in the submatrix for Reproductive traits (*r* = 0.60; *P* = 0.31), while the submatrices for Vegetative/Flowering Time (*r* = 0.93; *P* = 0.00) and Environmental Response traits (*r* = 0.91; *P* = 0.001) are well-conserved. We note that it is the changing variances in reproductive submatrix (i.e., the diagonal elements) that made the change in ***G***-matrix very strong, while the genetic covariances have changed less.

We observed the same trends when the four matching pairs of individual populations were analyzed separately (*SI Appendix*, Table S7). The teosinte and maize landrace ***G***-matrices are quite different in all four individual populations (*r* = 0.14 to 0.25; *P* = 1.00), primarily due to changes in the submatrix for Reproductive traits, while the submatrices for Vegetative/Flowering Time and Environmental Response traits are generally more conserved. We conclude that the ***G***-matrices of teosinte and maize have diverged primarily due to changes in the submatrix for reproductive traits, a conclusion consistent with the results of Yang et al. (6).

#### The *G*-matrix in teosinte placed considerable constraint on selection during the early domestication process.

We measured the degree of evolutionary constraint by calculating ***θ_T_***, the angle between the actual domestication trajectory (***Z***) and the teosinte genetic line of least resistance (***g_max,T_***). ***Z*** is a vector of difference in trait means between teosinte and maize landrace that represents the multitrait selection response, while ***g_max,T_*** is the first leading eigenvector of teosinte ***G***-matrix that accounts for 26.1% of the trait variance within teosinte. ***θ_T_*** has a possible range from 0° to 90°, where a small ***θ_T_*** means that evolution is least constrained (because the multitrait response to selection is in a similar direction as the first eigenvector of ***G***), while a large ***θ_T_*** means that the evolution is strongly constrained (selection changed traits in a very different direction than the genetic covariances naturally point). Evolutionary constraint would slow trait evolution since selection on one trait can be counteracted by another due to unfavorable genetic correlation. Our estimate of ***θ_T_*** is 66.3°, which suggests that maize domestication shows strong constraint imposed by the ***G***-matrix. Our estimate of ***θ_T_*** is very similar to the ***θ_T_*** of 67.3° estimated by Yang et al. (6).

The constraint imposed by teosinte ***G***-matrix stays true when the four matching pairs of teosinte and maize landrace populations were analyzed separately. However, the amount of constraint varied among individual populations. Specifically, ***g_max,T_*** explains 30.7%, 33.2%, 32.8%, and 26.3% of the variance, and the estimate of ***θ_T_*** is 83.9°, 60.7°, 75.9°, and 65.5° for Pop1, Pop2, Pop3, and Pop4, respectively.

#### In addition to the overall constraint indicated by the difference between *Z* and *g_max,T_*, we assayed how individual traits contributed to genetic constraint.

**Fig. 6.**
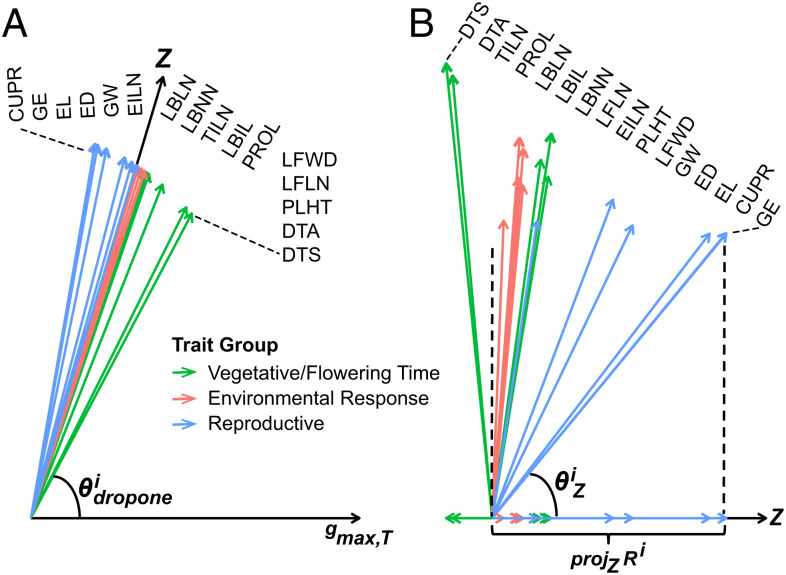
Constraints and consequences of multivariate selection. (*A*) Individual trait contribution toward genetic constraint is identified by dropping the ith trait from Z (actual domestication trajectory) and $g_{\max,T}$ (genetic lines of least resistance) and measuring the angle $\theta_{\mathrm{dropone}}^{i}$ between the two vectors. If $\theta_{\mathrm{dropone}}^{i}$ is smaller than $\theta_{T}$ = 66.3° (angle between Z and $g_{\max,T}$), then the ith trait is said to constrain evolution. If $\theta_{\mathrm{dropone}}^{i}$ is larger than $\theta_{T}$, then the ith trait is said to assist evolution. (*B*) Multivariate response ($R_{i}$) from hypothetical selection on a single ith trait. $R_{i}$ is compared to Z through the angle (${\theta^{i}}_{Z}$), and scalar projection ($\mathrm{pro}j_{Z}R^{i}$) of $R_{i}$ on Z. ${\theta^{i}}_{Z}$ measures the deviation from Z by selecting on the ith trait. $\mathrm{pro}j_{Z}R^{i}\;$ measures the evolutionary gain along Z by selecting on the ith trait.

By dropping one trait at a time and calculating the angle ***θ^i^_dropone_*** between ***Z_i_*** and ***g_max,T,i_***, we estimated individual trait contribution to genetic constraint (Fig. 6*A* and *SI Appendix*, Table S8). Trait *i* is considered to constrain evolution if the genetic constraint decreases (***θ^i^_dropone_*** < ***θ_T_***) after dropping trait *i*, while trait *i* assisted evolution if ***θ^i^_dropone_*** > ***θ_T_***. Our results showed that evolution was largely hindered by genetic correlations involving Vegetative/Flowering Time traits, but assisted by the genetic correlations involving Reproductive traits. This result is consistent with the result of Yang et al. (6). Although the genetic correlations among reproductive traits assisted evolution, genetic correlations between reproductive traits, which ancient farmers sought to improve, and flowering time, which were not direct targets of domestication, slowed the domestication process.

The drop-one analysis was also performed in the four matching pairs of individual populations separately. We observed similar results that Vegetative/Flowering Time ***G***-matrix constrained evolution, while Reproductive ***G***-matrix assisted evolution in all four individual populations (*SI Appendix*, Figs. S7–S10 and Table S9). These results are again consistent with the observations from Yang et al. (6).

#### The evolutionary constraint imposed by the *G*-matrix increased from teosinte to maize (*θ_M_ > θ_T_*) during domestication.

To compare how the amount of constraint changed during domestication, we also estimated ***θ_M_***, the angle between the domestication trajectory (***Z***) and the direction of maximum genetic variation in maize (***g_max,M_***). ***g_max,M_*** explains 22.3% of the variance in maize, and ***θ_M_*** is 80.4°, compared to 26.1% of the variance in teosinte explained by ***g_max,T_*** and ***θ_T_*** equal to 66.3°. The comparison between ***θ_M_*** and ***θ_T_*** suggests that there was substantial constraint in early domestication, and it increased over time. An increase of ***θ_M_*** compared to ***θ_T_*** would be expected if there was fixation of alleles that confer favorable genetic correlations during domestication, causing the structure of the ***G***-matrix to be more strongly affected by alleles that confer unfavorable correlations.

#### Selection on some individual traits would maximize the evolutionary gain along the domestication trajectory more than others.

Applying the multivariate breeder’s equation of ***R*** = ***Gβ***, we estimated the multivariate response (***R***) based on teosinte ***G***-matrix and hypothetical selection differentials (**β**). We used 16 different **β** vectors, with each ***β^i^*** having 1 element of a value of one and 15 elements of a value of zero. The ***i***th trait with a value of one in ***β^i^*** would be directly selected, while traits with a value of zero in ***β^i^*** are only indirectly selected. We obtained ***R^i^*** for each ***β^i^*** and then compared ***R^i^*** to the actual domestication trajectory ***Z*** for each ***i***th trait by calculating the angle between the two vectors (***θ^i^_Z_***) and also the scalar projection of ***R^i^*** on ***Z*** (***proj_Z_R^i^***) (Fig. 6*B* and *SI Appendix*, Table S10). ***θ^i^_Z_*** can range from 0° to 180°, where a larger angle means larger deviation of ***R_i_*** from ***Z***. This allowed us to evaluate to what extent selection on any one trait would maximize the evolutionary gain along the domestication trajectory for all traits. Among the 16 traits, GE, CUPR, and EL have the smallest ***θ^i^_Z_*** and the largest ***proj_Z_R^i^*** and also the largest ***θ^i^_dropone_***, indicating that selection for GE/CUPR/EL gives an overall response most closely aligned with the evolutionary trajectory, as well as the most evolutionary gain along the trajectory, and contributed less than others to genetic constraint. We observed similar results when the four matching pairs of individual populations were analyzed separately (*SI Appendix*, Figs. S7–S10 and Table S11). One exception is that LBNN has the smallest ***θ^i^_Z_*** and largest ***proj_Z_R^i^*** in Pop1. However, LBNN also has the smallest ***θ^i^_dropone_*** in Pop1. Taken together, our results confirm the conclusion of Yang et al. (6) that if the ancient farmers were to domesticate teosinte by selecting for a single trait, any of GE, CUPR, or EL would be ideal, as they had the maximum desired multivariate gains with the least genetic constraint. Furthermore, these three traits are themselves positively correlated through pleiotropy, as increasing ear length increases the capacity of the ear for higher cupules per row and grains per ear.

### Diversity of Genetic Architecture within Teosinte and Maize.

#### The overall amount of heritable variation (*h^2^*) for traits is generally conserved among teosinte populations and among maize landrace populations.

We compared *h*^2^ of the 16 traits for teosinte and 18 traits for maize landrace in the separate individual populations. We observed a similar level of *h*^2^ among teosinte populations, with 0.30, 0.34, 0.34, and 0.34 in Pop1, Pop2, Pop3, and Pop4 (*SI Appendix*, Fig. S2). Among the three trait groups, Reproductive traits showed the highest *h*^2^ in three out of four teosinte populations, followed by Vegetative/Flowering Time traits and Environmental Response traits. Of the genetic variance components, the proportion of additive genetic variance (heritable variation) predominates in all four teosinte populations, but among the three trait groups, the proportion of dominance variance is larger for Environmental Response traits. Similarly, we observed similar levels of *h*^2^ among maize landrace populations, with 0.16, 0.14, 0.16, and 0.19 in Pop1, Pop2, Pop3, and Pop4 (*SI Appendix*, Fig. S3). However, among the three trait groups, Vegetative/Flowering Time traits showed the highest *h*^2^ in all four maize landrace populations, followed by Environmental Response traits and Reproductive traits. For maize, there is more additive genetic variance (heritable variation) than dominance variance overall and for Vegetative/Flowering Time traits, but the proportion of dominance variance is much larger for Environmental Response traits and Reproductive traits. These results suggest that the additive genetic variance was reduced during maize domestication, resulting in a consequential increase of dominance variance in maize.

#### The genetic correlations among traits are conserved among populations within teosinte and within maize landrace, with the strongest conservation for Reproductive traits.

By pairwise comparison using the Mantel test, we observed that the genetic correlations (***r_g_***) among the four small teosinte populations are significantly correlated with each other (*r* = 0.47 to 0.81; *P* < 0.0001), with the strongest conservation observed within the Reproductive trait group (*r* = 0.98 to 0.99; *P* = 0.003 to 0.006) (*SI Appendix*, Table S12). The eigenstructure comparison also showed that the Reproductive trait group differs little in genetic correlations among the four small teosinte populations (*SI Appendix*, Table S12). Among the comparisons, Pop1 shows lower genetic correlation overall with the other three teosinte populations, while Pop2, Pop3, and Pop4 show higher genetic correlation with each other.

Similarly, the genetic correlations (***r_g_***) among the four maize landrace populations are also significantly correlated with each other (*r* = 0.39 to 0.60; *P* < 0.0001), although not as highly correlated as the four teosinte populations (*SI Appendix*, Table S13). Overall, the genetic correlations are consistent within the Reproductive trait group, but there is a wider range of variation in genetic correlations among the different trait groups for the four maize landrace populations. Among these comparisons, Pop1 and Pop2 differ most in genetic correlations among the four small landrace populations.

We also compared the genetic correlations of the four combined teosinte populations and the four combined maize populations with the single large teosinte and the matched single large maize landrace population reported by Yang et al. (6) (*SI Appendix*, Table S14). The Mantel test showed that the genetic correlations are strongly conserved for teosinte (*r* = 0.93; *P* < 0.0001), but more moderately conserved for maize landrace (*r* = 0.71; *P* < 0.0001). For teosinte, the strongest conservation was observed within the Reproductive trait group (*r* = 0.98; *P* = 0.005), followed by the Environmental Response trait group (*r* = 0.95; *P* = 0.02) and the Vegetative/Flowering Time trait group (*r* = 0.92; *P* = 0.03). For maize landrace, the strongest conservation was observed within the Vegetative/Flowering Time trait group (*r* = 0.93; *P* = 0.02), followed by the Environmental Response trait group (*r* = 0.91; *P* = 0.02) and the Reproductive trait group (*r* = 0.81; *P* = 0.002). The eigenstructure comparison also showed that the Reproductive trait group differs very little in genetic correlations for teosinte, with the first two leading eigenvectors only 2.2° and 3.4° apart, while the Vegetative/Flowering Time trait group differs little for maize landrace, with the first two leading eigenvectors 14.3° and 9.4° apart.

#### The *G*-matrices are highly similar within teosinte and within maize landrace.

We also tested the similarity of ***G***-matrices among the four small teosinte populations or the four maize landrace populations by comparing the predicted evolutionary responses using Random Skewers (*SI Appendix*, Table S15). The predicted evolutionary responses among different teosinte populations, both overall (*r* = 0.88 to 0.98; *P* = 0.00) and within different trait groups (*r* = 0.87 to 0.99; *P* = 0.00), are significantly more correlated than random, suggesting that the ***G***-matrices of the four individual teosinte populations are highly similar. This result may suggest that there would be little difference if one started domestication with a different teosinte population. Similarly, the predicted evolutionary responses among different maize landrace populations (*r* = 0.61 to 0.83; *P* = 0 to 0.003) are also significantly more correlated than random, suggesting that ***G***-matrices of the four individual landrace populations are similar, but with exceptions between Pop2 and Pop3 (*r* = 0.51; *P* = 0.25). This result also indicates that maize landrace populations exhibit more among population diversity than teosinte populations. Overall, our data suggest that there is far less difference in ***G***-matrices among populations within teosinte or maize landrace than that between teosinte and maize landrace.

#### Variation among populations within teosinte or maize is to some extent driven by selection, but much weaker than selection between teosinte and maize.

**Fig. 7.**
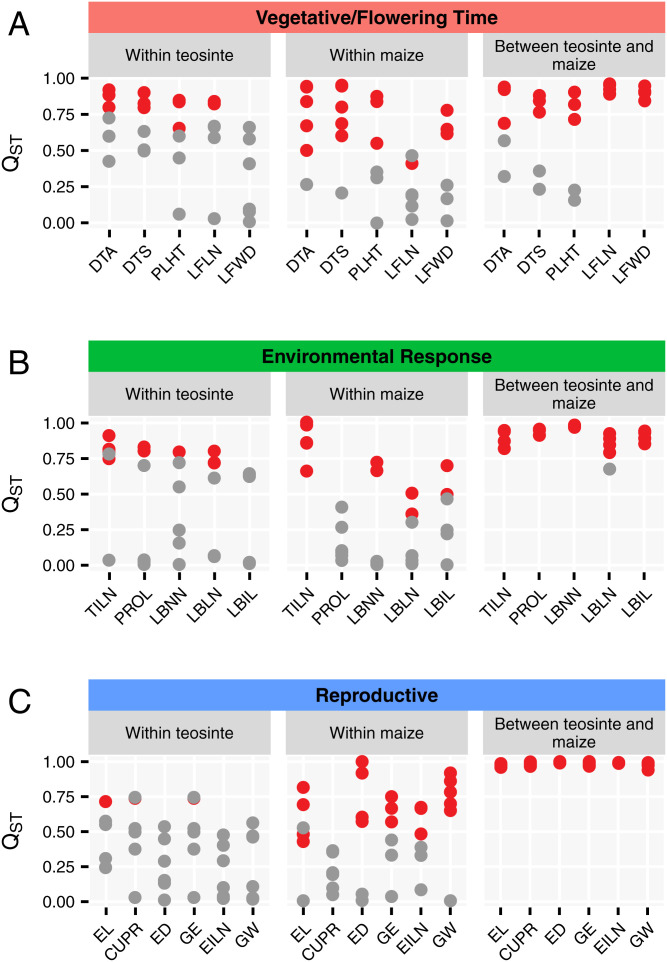
Summary of *Q_ST_* within teosinte and maize and between teosinte and maize. Within teosinte or maize, *Q_ST_* was estimated in a pairwise comparison of teosinte or maize populations. Between teosinte and maize, *Q_ST_* was estimated in both combined and each pair of teosinte and maize populations. The three trait groups are shown in *A–C*. Significant *Q_ST_* by univariate *Q_ST_*–*F_ST_* tests are shown in red dots, while nonsignificant *Q_ST_* are shown in gray dots.

Above, we show that selection was a strong force to drive the difference of phenotype between maize and teosinte through *Q_ST_*–*F_ST_* tests, especially for Reproductive traits, but also for Environmental Response traits, and fairly strong for Vegetative/Flowering Time traits. Now, we ask if variation among populations within teosinte or maize is similarly driven by selection. We performed univariate *Q_ST_*–*F_ST_* tests for individual traits in pairwise comparison of teosinte or maize landrace populations to see whether neutral evolution can be rejected (*SI Appendix*, Tables S16 and S17). Among teosinte populations, 11 out of 30 tests (*Q_ST_* = 0.65 to 0.92) for Vegetative/Flowering Time traits, 11 out of 30 tests (*Q_ST_* = 0.63 to 0.91) for Environmental Response traits, and 3 out of 36 tests (*Q_ST_* = 0.72 to 0.74) for Reproductive traits reject the null hypothesis of selective neutrality (*P* < 0.05) (Fig. 7). Among maize, 17 out of 30 tests (*Q_ST_* = 0.41 to 0.95) for Vegetative/Flowering Time traits, 13 out of 30 tests (*Q_ST_* = 0.36 to 1) for Environmental Response traits, and 18 out of 36 tests (*Q_ST_* = 0.43 to 1) for Reproductive traits reject the null hypothesis of selective neutrality (*P* < 0.05) (Fig. 7). Between maize and teosinte, 19 out of 25 tests (*Q_ST_* = 0.69 to 0.96) for Vegetative/Flowering Time traits, 24 out of 25 tests (*Q_ST_* = 0.79 to 0.98) for Environmental Response traits, and 30 out of 30 tests (*Q_ST_* = 0.94 to 1) for Reproductive traits reject the null hypothesis of selective neutrality (*P* < 0.05) (Fig. 7). These results suggest that there is some evidence of selection for Vegetative/Flowering Time traits and Environmental Response traits among teosinte populations or among maize populations, but it is not as strong as between maize and teosinte. For Reproductive traits, there is virtually no evidence for selection driving differences within teosinte, but some evidence for selection within maize. These results might reflect that farmers were selecting for different ear types in different locations, but reproductive traits did not contribute to local adaptation in teosinte.

## Discussion

Despite decades of genetic research on domestication, the tools of evolutionary quantitative genetics have only been sparsely utilized to interrogate how domestication was constrained by the genetic architecture of the wild ancestor and how genetic architecture was altered in the crop species. The work reported by Yang et al. (6) is unique in the domestication literature for reporting the first results on how the genetic architecture influenced and was altered by maize domestication, but the study was based on a single large teosinte population and a matching single large maize landrace population. If genetic architecture for the key domestication, adaptation, and fitness traits measured by Yang et al. (6) varies significantly among populations within each subspecies, the population samples may not be representative of teosinte and maize more broadly. In this study, we tested if the results from Yang et al. (6) would be confirmed with a broader sample of teosinte and maize landrace populations. Using a diverse sample of four teosinte populations and four matching maize landrace populations, we assay the structure of genetic variances, genetic correlations among traits, strength of selection during domestication, and diversity in genetic architecture with teosinte and maize.

### Confirming Genetic Architecture of Maize Domestication with a Broad Sample.

Yang et al. (6) observed a drop in additive genetic variance and an increase in dominance genetic variance following maize domestication. Furthermore, the genetic architecture of adaptation was reconfigured such that the relative importance of genetic-by-environment interaction (G×E) variance for Vegetative/Flowering Time traits and Environmental Response traits decreased and for Reproductive traits increased in maize compared to teosinte. The genetic correlations are moderately conserved among traits between teosinte and maize, with the strongest conservation within Vegetative/Flowering Time traits. While there is some conservation in the genetic correlations, the ***G***-matrices of teosinte and maize landrace are quite different, primarily due to changes in the submatrix for Reproductive traits. Our analyses confirmed all of these results both by comparing our four teosinte populations to their four matched maize populations individually and by an analysis of the combined teosinte and maize populations.

The decrease in additive genetic variance consistently observed in maize compared to their sympatric teosinte populations can be attributed to the population bottleneck and selection during maize domestication. Maize experienced a mild genetic bottleneck, as domesticated maize retained ∼81% of the genetic diversity of its wild ancestor, teosinte (10). The genetic diversity of ∼2 to 4% of maize genes targeted by selection was further reduced beyond that caused by the domestication bottleneck (10, 11). Maize also experienced a loss of gene-expression variation due to selection on *cis*-regulatory differences during domestication (12). Thus, many functional alleles were lost or brought to fixation during maize domestication, a result consistent with our observation that additive genetic variance at the level of domestication trait phenotypes has also been reduced.

The changes in the relative apportionment of G×E variance during domestication may indicate that teosinte and maize may have evolved different strategies to cope with environmental challenges. Teosinte plants, which have multiple long lateral branches that bear many small ears, adapted to a wide range of environmental challenges in the wild by modulating branching and ear number per plant. Long branches with many ears optimize seed production in resource-rich environments. In resource-poor environments, short branches and few ears still enable the plant to reproduce. The number of grains per ear and grain weight are held constant. Accordingly, teosinte exhibits higher G×E variance for Vegetative/Flowering Time traits and Environmental Response traits than maize. By contrast, the maize plant has a single stalk with few short branches typically bearing single large ears in both resource-rich and -poor environments. Thus, maize responds to environmental vagaries by changes in grains per ear and grain weight, as revealed by the higher G×E variance for Reproductive traits.

The conservation of genetic correlations and dissimilarity of ***G***-matrices indicate that Reproductive traits experienced stronger selection than other domestication traits during maize domestication. Yang et al. (6) also observed that selection intensities (*i*) during domestication were generally weak and within the lower range of values observed under natural selection (7). Consistent with expectations based on the changes in phenotype between maize and teosinte, Reproductive traits showed the highest values of *i* during maize domestication, and Vegetative/Flowering Time traits and Environmental Response traits showed much lower values. Again, we confirmed the observations both by the analysis of our four separate paired populations individually or the combined populations. We rejected virtually all hypotheses of neutrality for traits at different levels through *Q_ST_*–*F_ST_* tests. These results indicate that domestication is a process of slow evolution, and maize domestication largely focused on selection for Reproductive traits. Previous archaeological data also revealed slow rates of phenotypic evolution during plant domestication, which are significantly lower or comparable to those observed among wild species subjected to natural selection (13).

### Variation of Genetic Architecture among Populations of a Single Species.

We observed that the amount of heritable variation is roughly equal among teosinte populations, but among populations in other species, this is not always the case. The heritability for leaf number, leaf thickness, and reproductive stage changed widely among three populations of the native annual legume *Chamaecrista fasciculate* across three geographic ranges, indicating adaptive evolution in response to global warming (14). Estimates of the additive genetic variance for fitness also varied widely among three wild populations of annual legume *C. fasciculata* over 3 y (15). Fall-germinating plants have a much higher heritability than spring-germinating plants within an annual population of *Mimulus guttatus* (16). The wider variation in heritability among populations of other wild species than we observed in teosinte suggests that our teosinte populations have a relatively conserved genetic architecture for domestication traits.

We observed that both the genetic correlation matrices and the ***G***-matrices are well-conserved among teosinte populations. We asked whether such conservation is observed among populations in other species. In response to global warming, genetic correlations between leaf number, leaf thickness, and reproductive stage vary widely among three populations of the native annual legume *C. fasciculate* across three geographic ranges (14). Genetic correlations between germination, growth, and flowering traits changed significantly between the fall and spring cohorts in an annual population of *M. guttatus* (16). The ***G***-matrices among flowering traits are similar among natural populations of *Arabidopsis lyrata* (17). O’Brien et al. (18) showed that teosinte populations (*Zea mays* ssp. *mexicana*) adapted to different rhizosphere biota across environment had significantly different ***G***-matrices. The ***G***-matrices for ear and flowering traits in maize landrace populations differed both within-village and among-villages (19). In general, the considerable differences observed among populations in these other studies suggests that our teosinte populations have a relatively conserved genetic architecture for domestication traits.

We observed a drop in heritable variance (*h*^2^) in the postselection maize populations, as compared to teosinte. Some research in other species suggests that selection may or may not change heritable variance. In a large wild bird population, preselection and postselection samples (from fledging to breeding) had virtually identical *h*^2^ for relative body weight, although the additive genetic variance itself was significantly lower in the postselection samples (20). For an Australian native wildflower (*Senecio pinnatifolius*), four contrasting ecotypes showed different *h*^2^ of plant architecture and leaf traits, indicating that heritability can change after adaptive divergence among ecotypes (21).

Among teosinte populations, we observed little evidence of selection on Reproductive traits, but modest evidence of selection for Vegetative/Flowering Time traits, by *Q_ST_*–*F_ST_* tests. Evidence for adaptive divergence among populations of other plant species appears stronger. In wild barley, different populations can be differentiated into three ecotypes (north, coast, and desert), and the three ecotypes diverged in morphological characteristics, as shown by *Q_ST_*–*F_ST_* tests (22). Similarly, among *scarlet gilia* populations, several floral traits show significantly greater *Q_ST_* than mean *F_ST_* (23). Phenotypic differentiation (*Q_ST_*) between geographically structured red and yellow floral races of *Mimulus aurantiacus* is much greater than neutral genetic differentiation (*F_ST_*) (24). In the silverleaf sunflower, divergent selection drove divergence in life history traits across the species range, where early flowering syndrome is primarily observed in populations from coastal barrier islands, while populations from the nearby mainland coast are primarily late flowering (25). Among maize landrace populations, *Q_ST_* values measured for ear and flowering traits are significantly higher than F_ST_ values among populations (19). *Q_ST_*–*F_ST_* tests also indicate strong divergences for metabolites between wild and cultivated species in maize, tetraploid wheat, and lettuce (26–28). Perhaps the cause of our somewhat weaker evidence for adaptive divergence among teosinte populations as compared to other wild species is that we assayed traits expected to be important in domestication, and not traits necessarily expected to drive adaption to variation in the natural environment.

Similar to our observation of a dramatic changes in ***G***-matrices following domestication, the ***G***-matrices for plant architecture and leaf traits changed significantly among four contrasting ecotypes of an Australian native wildflower (*S. pinnatifolius*), presumably due to selection (21). ***G***-matrices of important fitness traits are reported to be significantly different among and within species of other genera, including the flowering plant genus *Aquilegia* (29), three species of Gomphocerine grasshoppers (30), and among species of the genus *Lycaeides* butterflies (31). The ***G***-matrices of four different morphological traits in a natural bird population changed significantly over 25 y (32). In general, significant changes in ***G***-matrices are observed following selection, consistent with our results.

## Concluding Statement

Our analysis was primarily undertaken to test if the conclusions drawn by Yang et al. (6) based on the analysis of single teosinte and maize populations were consistent with more broadly representative samples of maize and teosinte. Our results indicate that those conclusions do indeed generalize to a more diverse set of populations. We also conclude that the differences in genetic architecture between teosinte and maize are not fundamentally different from those observed between populations of some natural species before and after selection. Finally, for domestication traits, genetic architecture is well-conserved among teosinte populations, indicating that any relatively large teosinte population could have served effectively as the starting point for the selective breeding program that produced the most dramatic change in morphology known between any crop species and its progenitor.

## Materials and Methods

### Populations.

We sampled four populations of teosinte (*Z. mays* ssp. *parviglumis*) and four populations of maize landraces (*Z. mays* ssp. *mays*) from the states of Michoacán, Guerrero, Nayarit, and Jalisco, respectively, in Mexico (*SI Appendix*, Table S18). Each maize landrace population was sampled at a location near the corresponding teosinte population. Each of the teosinte progeny populations was created by selfing and intermating 10 teosinte parent plants to produce progeny seed/plants. We also applied a similar crossing strategy to develop the four maize landrace progeny populations. We obtained 40 selfed and 284 outcross families for teosinte and 36 selfed and 134 outcross families for maize landrace (*SI Appendix*, *SI Materials and Methods*).

### Genotyping.

Leaf tissues from the parents and progeny of teosinte and maize landrace were collected for DNA extraction and genotyping by using Genotype-by-Sequencing (GBS) (33), by which DNA samples were digested using ApeKI restriction enzyme and sequenced in 96-plex on Illumina HiSeq 2000, SE 1 × 100 bp (Illumina). After that, GBS raw sequencing reads were processed for genotype calling by using the TASSEL5 GBS v2 Pipeline (34). Using GBS raw genotypes of the parents and progeny, we inferred parentage of each progeny for both teosinte and maize landrace as described (6). The CrossMap (35) software was used for uplifting the GBS SNP positions from AGPv2 to AGPv4. We then imputed the GBS data for teosinte and maize landrace using the ParentPhasingPlugin and ImputeProgenyStatesPlugin, as implemented in TASSEL5 (36). Details of SNP imputation and filtering can be found in *SI Appendix*, *SI Materials and Methods*. The missing data were imputed by using LD-KNNi, as implemented in TASSEL5 (36). We obtained final imputed data of 5,101 progeny and 48,128 sites for teosinte and 4,641 progeny and 53,277 sites for maize landrace (*SI Appendix*, Table S19). We estimated additive and dominance-realized genomic relationships for all progenies combined within each subspecies and also separately for all populations.

### Phenotyping.

The teosinte and maize landrace progenies were grown in adjacent plots near Homestead, FL, over two winter seasons (2015–2016 and 2016–2017) for field evaluations. Within each season, a randomized design was used for both teosinte and maize landrace, with individual plants as experimental units. We collected phenotypic data for a total of 16 traits from 5,101 teosinte progeny and 4,641 maize landrace progeny. The trait abbreviations can be found in Table 1, and the details of how we measured the traits can be found in Yang et al. (6).

### Univariate Analysis.

A common univariate linear mixed model was fit for each trait by using ASReml v4, which implements residual or restricted maximum-likelihood estimation of the variance parameters (37). We performed univariate analysis using the combined set of four teosinte populations and four maize landrace populations, separately. In the model, the fixed effects include population, year, inbreeding coefficient, shading, and field positions, and the random effects are polygenic additive, dominance, and genetic-by-environment effects, where the additive and dominance effects were modeled as having covariances proportional to the combined additive and dominance-realized relationship matrices, respectively. We also performed univariate analysis in each individual population for teosinte and maize landrace, using the population-specific realized relationship matrices in this case. Based on our univariate analysis results, we estimated the selection intensity (*i*) for each trait by applying the univariate breeder’s equation (38) as described (6). We assumed 4,500 or 9,000 generations of selection while calculating selection intensities. The full model and details can be found in *SI Appendix*, *SI Materials and Methods*.

### Multivariate Analysis.

A bivariate linear mixed model was fit by using ASReml v4 (37) for each pair of traits to estimate additive genetic covariances based on the additive genomic relationship matrices. Like univariate analysis, we performed multivariate analysis using the combined set of the four teosinte populations and four maize landrace populations separately. All of the fixed and random effects were the same as univariate analysis, except that we excluded polygenic dominance and genetic-by-environment effects as a compromise for improvement of computational speed. We also performed multivariate analysis in each individual population for teosinte and maize landrace. The full model and details are described in *SI Appendix*, *SI Materials and Methods*.

We tested the conservation of genetic correlation matrices between teosinte and maize landrace using the Mantel test (7). We also tested the conservation of genetic correlation matrices within teosinte or within maize landrace. The Mantel test is performed by using the *mantel.test* function with 10,000 permutations implemented in the *ape* package (39) in R. We also calculated the angle between the first two leading eigenvectors of two matrices as a complementary analysis for the Mantel test.

We applied Random Skewers (8) to compare the structure of G-matrices for teosinte and maize landrace, which tests for similarity between two matrices (G) by comparing the predicted evolutionary responses (R) calculated from the multivariate breeder’s equation of R=Gβ. This test is performed by using the *skewers* function with 1,000 simulations implemented in the *phytools* package (40) in R. To test whether neutral evolution can be rejected at individual trait level, we performed a univariate $Q_{ST}-F_{ST}$ test for each trait using R scripts provided by Whitlock and Guillaume (41).

We quantified the genetic constraint from the G-matrix using the angle (θ) between the genetic lines of least resistance ($g_{\max}$) (42) and the actual domestication trajectory (Z).$g_{\max}$ is the eigenvector of G that accounts for the most variation, while Z is a vector of difference in trait means between teosinte and maize landrace. To measure individual trait contribution to genetic constraint, we calculated the angle $\theta_{\mathrm{dropone}}^{i}$ between Z and $g_{\max}$ for each ***i***th trait that is dropped from Z and$g_{\max}$. Applying multivariate breeder’s equation of R=Gβ, we compared $R^{i}$ to the actual domestication trajectory Z for each ***i***th trait by calculating the angle between the two vectors ($\theta_{Z}$) and also the scalar projection of $R^{i}$ on Z ($\mathrm{pro}j_{Z}R^{i}$). More details of these analyses can be found in *SI Appendix*, *SI Materials and Methods*.

## Data Availability

Genotype and phenotype files used in this study are available in FigShare (https://doi.org/10.6084/m9.figshare.14983944). All study data are included in the article and/or *SI Appendix*.
